# Manufacturing of Al Alloy Microrods by Micro Cutting in a Micromachining Center

**DOI:** 10.3390/mi10120831

**Published:** 2019-11-29

**Authors:** M. Azizur Rahman, Mustafizur Rahman, Mozammel Mia, A.B.M.A. Asad, Ahmed Fardin

**Affiliations:** 1 Department of Mechanical and Production Engineering, Ahsanullah University of Science and Technology, Dhaka 1208, Bangladesh; azizur777@gmail.com or; 2 Department of Mechanical Engineering, National University of Singapore (NUS), 10, Kent Ridge Crescent, Singapore 119260, Singapore or mustafizur.rahman23@gmail.com (M.R.); or; 3 Department of Mechanical Engineering, Imperial College London, London SW7 2AZ, UK; 4 Centre for Sustainable Development, Department of Engineering, University of Cambridge, Cambridge CB2 1PZ, UK or

**Keywords:** micromachining, aluminum (Al) alloy, microrods, microchips, micro components

## Abstract

This paper presents the micromanufacturing of aluminum (Al) alloy microrods using micro turning as a competing process to other nontraditional micromachining methods. In that regard, the challenges in such manufacturing have been identified and overcome. The strategies of step-by-step cutting have also been delineated. In addition, the influence of step size and step length on the cutting and thrust forces were investigated. The chip morphology for micromachining was examined using scanning electron microscopic imagery. The safe dimension of the microrod was calculated and, subsequently, used to fabricate microrod, conical tip rod, and grooved rod from 3 mm long and 1.5 mm diameter rod using an appropriately coded computer numerical control (CNC) micromachining center. Our results showed that the thrust force was responsible for part deflection, emphasizing the necessity for computing safe dimensions. At shallow step sizes, the thrust force was more dominant, causing plastic deformation associated with rubbing and burnishing. The chips produced were irregular and sliced in nature. Conversely, at high step sizes, the cutting force superseded the thrust force, resulting in chips that were spread more along the width as opposed to the depth. The chips also had a smoother interacting surface. Finally, micro turning was successfully implemented to manufacture milli-scale structures (i.e., 3 mm long) with micro features (150 to 230 μm diameter) on aluminum alloy materials.

## 1. Introduction

Driven by the rising demand for microsized components and products, miniaturization has experienced rapid growth in recent times. With the rise of manufacturing accuracy, much progress has also been made in the development of engineered microsized components. Micromachining is leading the advancements in the micromanufacturing domain by narrowing the gap between conventional machining and microelectromechanical system (MEMS) technology. Micromachining, as the name suggests, refers to the production of parts in the micron regime, within the 1 μm to 999 μm range, by machining or removal of material. However, a limit has been placed recently that confines micromachining to below the 500 μm bound, distinguishing it distinctly from macromachining.

One particular miniature part, the micro rod electrode, is rapidly gaining popularity in various micromachining and measurement applications. For example, processes such as micro electro-discharge machining (μ-EDM) and micro drilling use these microrods and microshafts as microelectrodes or micro cutting tools to perform machining operations such as micro holes in fuel injection nozzles [1]. Also, such microrods are increasingly being used as metallic needle probes to detect and measure the properties of electronic devices [2]. The challenge of manufacturing these microsized parts arises from the increment of the surface area to volume ratio as the size decreases. Nevertheless, there are various manufacturing processes that rise up to the challenge.

**Figure 1:**
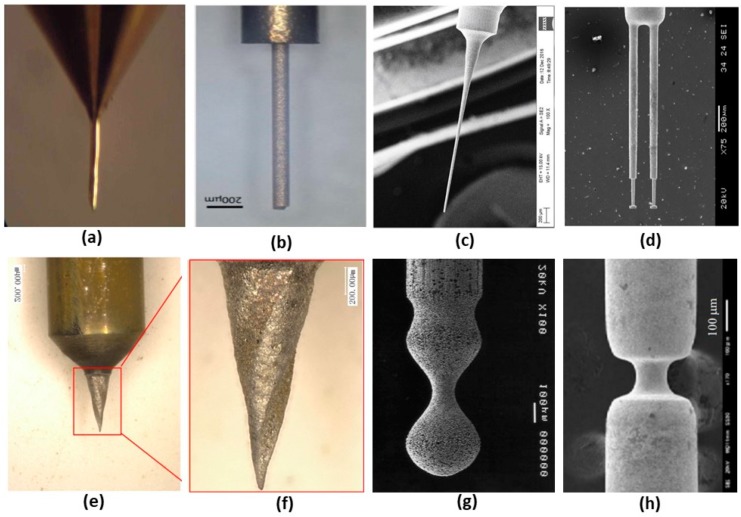
Various shapes of microrods produce by (**a**) conventional micro turning, (**b**) micro electrical-discharge grinding (μ-EDG), (**c**) micro controlled inclined grinding (μ-CIG), (**d**) microelectrochemical machining (μ-ECM) + micro reverse electro-discharge machining (μ-REDM), (**e,f**) Low-speed micro wire electrical-discharge turning (LS-μWEDT), (**g**) electrolyte jet machining (EJM), and (**h**) micro milling + electrochemical turning in EJM.

Conventional micro turning technology can be applied to produce microshafts [3] as shown in Figure 1a. In this paper, this technology is explored further. Next, microrods can also be manufactured using the micro wire electro-discharge grinding (μ-WEDG) and micro block electro-discharge grinding (μ-BEDG) processes. However, the on-process measurements limit the ability of μ-WEDG and μ-BEDG to accurately shape the microrods. To eliminate this issue, the electrical-discharge grinding (μ-EDG) process can be adopted. A 106 μm diameter micro rod electrode manufactured by deploying two block-electrodes (μ-EDG-TBE) is shown in Figure 1b [4].

On the one hand, such nontraditional machining, especially by discharge machining, is typically time-consuming. On the other hand, microtool manufacturing using the faster traditional mechanical machining, usually by a specialized processing machine, is usually costly. Thus, controlled inclined grinding (CIG) was developed to produce microrods simply, cheaply, and efficiently. A 20 μm diameter tool produced using CIG is shown in Figure 1c [5].

Electrochemical machining (ECM) can also be used for this micromanufacturing purpose. In one example, ECM, particularly the electrolysis machining, was deployed to fabricate a 100 μm micro pin used for a scanning tunneling microscope (STM) [6]. However, the rotation speed of the workpiece posed a threat to accurate machinability. For example, even though a cylindrical microelectrode was achieved at a rotational speed of 1000 rpm, the shape deformed to conical at 1500 rpm. Moreover, over 2000 rpm, the workpiece showed both shape deformity and surface scrapes. This issue has been addressed by combining micro-ECM with micro reverse electro-discharge machining (μ-REDM) to improve productivity and efficiency. A dual disc-type tipped microelectrode [7] produced using the combined process is shown in Figure 1d.

Low-speed wire electrical discharge turning (LS-WEDT) is another process that can be adopted to confront the micromanufacturing challenges. Figure 1e,f shows complex tapered microelectrodes with spirals fabricated using LS-WEDT [8]. Similar complex shapes can be achieved through electrolyte jet machining (EJM). A profiled microrod manufactured using EJM by synchronizing the machining current and the motion of scanning is shown in Figure 1g [9]. Micro grooving on microshafts like the one shown in Figure 1h can be achieved by combining micro milling and electrochemical turning [10].

Returning to the topic of micro turning, it is a scaled-down version of the conventional turning process which is able to manufacture three-dimensional (3D) micro features using a solid cutting tool. As micro turning is a tool-based process, the tool path can be defined clearly, and hence the process output is deterministic to produce accurate 3D microsized parts. A 100 μm diameter micro shaft [3] produced using micro turning is shown in Figure 1a. However, the deflection of the thin workpiece is found to be the main problem associated with micro turning, which ultimately reduces the dimensional accuracy and surface integrity of the manufactured microrod. The various advantages and drawbacks of the manufacturing processes discussed above are summarized in Table 1.

**Figure 2:**
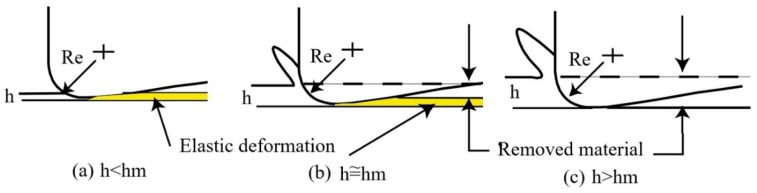
Schematic of chip formation in micro cutting and micro grinding Reproduced with permission from [16].

As observed, the majority of the processes for manufacturing microrods and microtools are nonconventional, especially discharge machining, and require cutting fluids that are harmful to the environment. Therefore, dry or near-to-dry cutting is gaining popularity over the years. One of several beneficial outcomes of dry machining is the improved environmental performance from the elimination of the cutting fluids [15]. Moreover, the principals of tool-based micromachining are similar to those of conventional macro cutting. Thus, an understanding of microchip formation is an utmost necessity to predict the micro cutting process. The concept of the chip formation is illustrated in Figure 2 [16]. From the schematic relationship of minimum chip thickness (*h*_m_) and actual chip thickness (*h*), it is inferred that chip formation will not occur unless the chip thickness reaches a certain value for material removal during micromachining and micro grinding. In this respect, ultraprecision machining, including turning, milling, grinding, and polishing, is utilized for chip removal in microscale to manufacture the high quality microstructured components [17].

**Figure 3:**
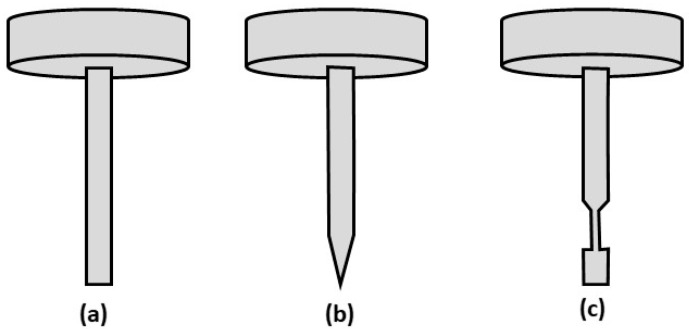
Shape of the microrods with (**a**) straight (**b**) conical and (**c**) grooved end.

Therefore, in this study, a conventional dry micro cutting using the micro turning method was adopted to manufacture the microrods (illustrated in Figure 3) from 3 mm long and 1.5 mm diameter for the initial workpiece. The mitigation of the workpiece deflection issue that is encountered in micro turning is a major exploratory topic in the study. Aluminum alloy is chosen as the workpiece for widespread use of aluminum alloys in the manufacturing industry owing to its superior corrosion resistance, high ratio of strength-to-weight, and high conductivity of heat and electricity.

## 2. Micro Turning Process Development

### 2.1. Modeling of the Machining Process

**Figure 4:**
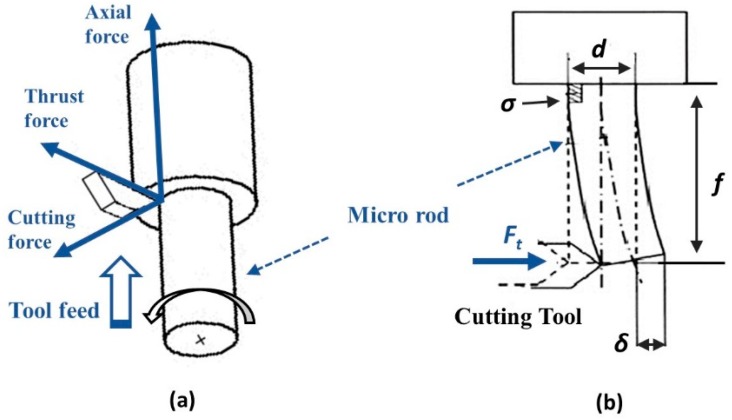
(**a**) Cutting force components and (**b**) workpiece deflection in micro turning [18].

During the micro turning operation, as the workpiece (microrod) becomes thinner, the rigidity of the microrod wanes causing it to be easily deflected by the thrust force of the cutting tool (Figure 4). This workpiece deflection can be minimized by curtailing the thrust force (*F*_t_).

If the diameter of the microrod is *d*, the force acting on the unsupported end of the microrod can be denoted as *F*_t_. In this condition, the microrod deflection (*δ*) and the resulting bending stress (*σ*) is determined by using the bending Equations (1) and (2), respectively [18].

(1) $$\delta =\frac{F_{t}f^{3}}{3EI}=\frac{64F_{t}f^{3}}{3\pi Ed^{4}}$$

(2) $$\sigma =\frac{32F_{t}f}{\pi d^{3}}$$

**Figure 5:**
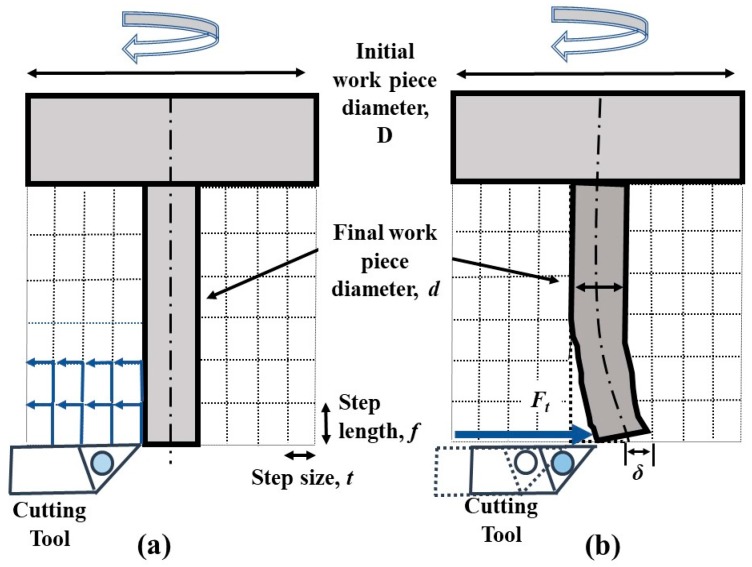
(**a**) Step cutting scheme and (**b**) workpiece deflection.

Microrods are manufactured by applying the micro cutting mechanism of the micro turning process in a stepwise scheme, as shown in Figure 5a. The stepwise maneuver helps to avoid the workpiece deflection shown in Figure 5b. The turning is performed in steps where the step size (*t*) is defined by the cutting depth in the radial direction. The step length (*f*), at which the rod will not undergo permanent deformation, is calculated from Equations (1) and (2).

If the *F*_t_ for a specific workpiece dimension (*d*) is known, the deflection (*δ*) and the resulting stress (*σ*) can be obtained from the above-mentioned equations. The limiting stress experienced by the workpiece should be restricted below the plastic deformation level. To achieve this, the thrust force must be kept below the estimated maximum value.

### 2.2. CNC Program Generation for Micromachining

**Figure 6:**
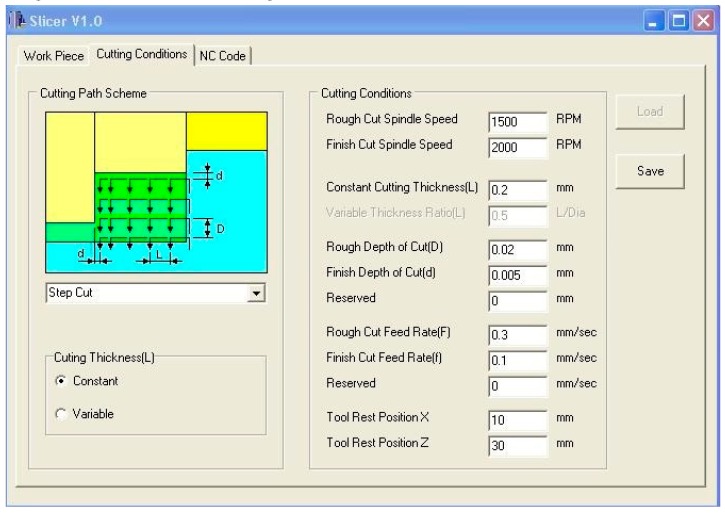
GUI (graphical user interface) for step cutting process in micro turning.

Precise control of the axis of a micromachine, with positioning error of less than 1 µm, is necessary to obtain micron-range machining dimensions [19]. Hence, the step cutting process of the microrod requires bespoke lines of numerical control (NC) codes that are not available in the usual computer-aided manufacturing (CAM) software. Thus, the SLICER program was developed, using C++ (Borland, Austin, TX, USA), to generate the CNC program (NC Codes) for the step cutting process by loading the workpiece profile and selecting the appropriate cutting parameters. Figure 6 shows the graphical user interface (GUI) of SLICER for the rough cutting condition with 1500 rev/min of spindle speed, 0.02 mm cutting depth (step size), 0.3 mm/s feed rate, and 0.2 mm cutting thickness (step length). The finish cutting conditions are as follows: 2000 rev/min spindle speed, 0.005 mm cutting depth (step size), and 0.1 mm/s feed rate. The *X* and *Z* axes tool rest positions are 10 mm and 30 mm, respectively. The NC codes, generated using the cutting conditions, were uploaded to the user interface of the micromachining center to run and perform the machining operation. Subsequently, the proposed microrod with straight end, as shown in Figure 3a, was fabricated using the step cutting scheme illustrated in Figure 5a.

**Figure 7:**
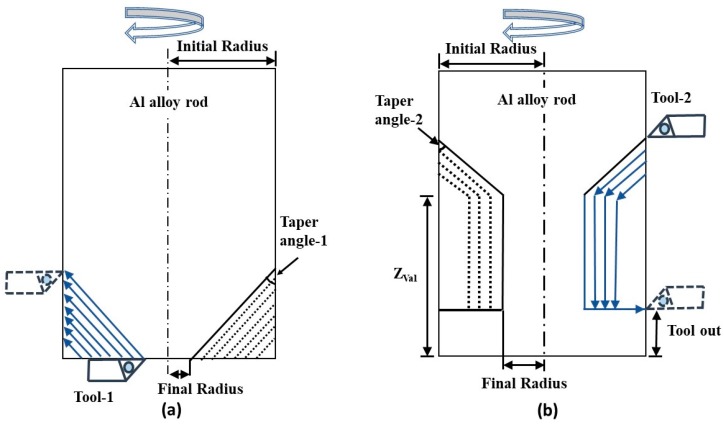
Micro taper turning scheme in (**a**) forward cutting and (**b**) reverse cutting.

The conical end of the microrod shown in Figure 3b was generated by taper micro turning. In this approach, the forward cutting is performed by moving the tool parallel to the tapered surface while rotating the workpiece. The approach is illustrated in Figure 7a. The grooved end of the microrod shown in Figure 3c was fabricated by combining the step cutting and taper turning process for a reverse cutting scheme. This is illustrated in Figure 7b.

**Figure 8:**
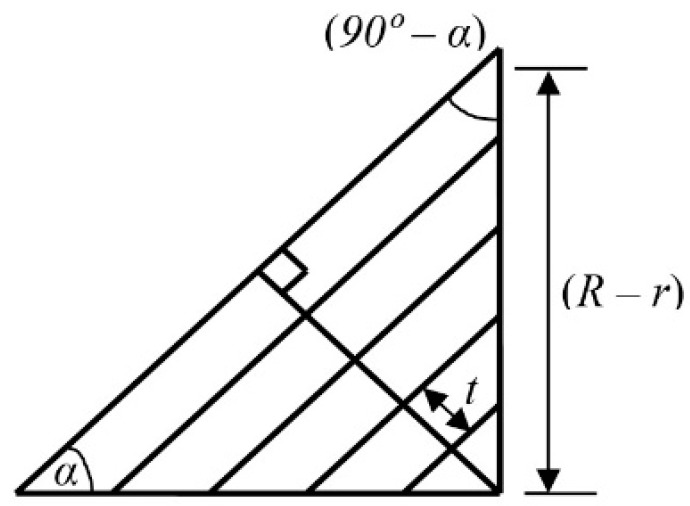
Schematic diagram of calculation of the number of cuts parallel to taper surface.

For step size (*t*), taper angle (*α*), initial taper radius (*R*) and final taper radius (*r*), the required rough cuts parallel to the tapered surface (*n*_t_) are calculated from Figure 8 using Equation (3).

(3) $$n_{t}\times t=\left(R-r\right)\sin\left(90-\alpha \right)$$

$$\mathrm{or},\;n_{t}=\left(R-r\right)\cos\alpha$$

Equation (3) was used as the governing equation in writing the source codes in C++ (Borland) to develop the NC program generator for taper turning. The NC codes for taper turning are generated according to the cutting path schemes (forward or reverse cut) using the dimensions of the workpiece and appropriate machining parameters. Finally, the generated NC codes (CNC program) are uploaded to the machine controller to run the machining operation.

## 3. Micromachining Experiment

### 3.1. Machining Center

**Figure 9:**
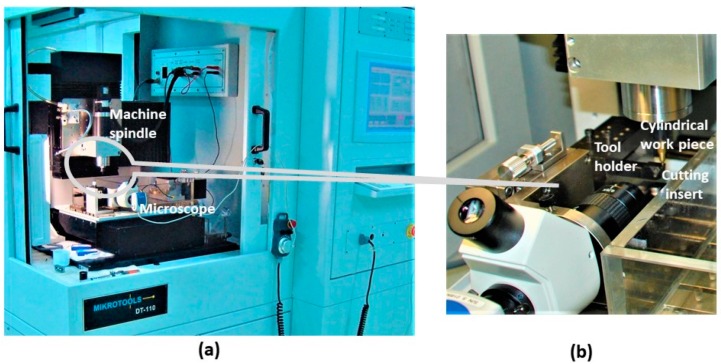
Arrangement of the (**a**) micromachining center, DT-110, (**b**) workpiece cutting tool holder and insert for micro turning.

Micromachining tests were conducted with a micromachining center (Mikrotools DT-110, Mikrotools Pte. Ltd., Singapore), shown in Figure 9, developed for micromachining with movements in three directions. The optical linear scale for each axes *X*, *Y*, and *Z*, has 0.1 µm resolution with closed-loop feedback control.

### 3.2. Material and Cutting Tool

**Figure 10:**
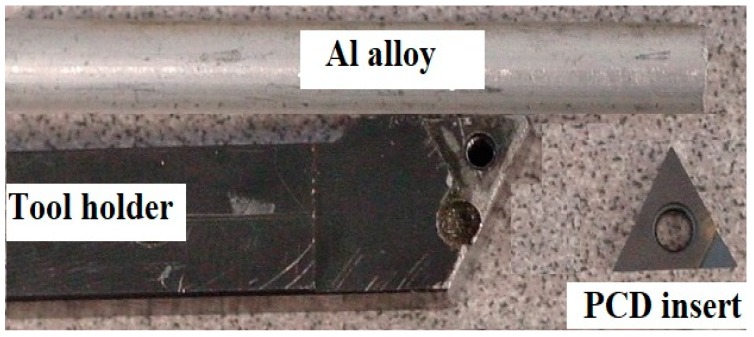
Aluminum (Al) alloy workpiece and polycrystalline diamond (PCD) cutting insert for micro turning.

**Figure 11:**
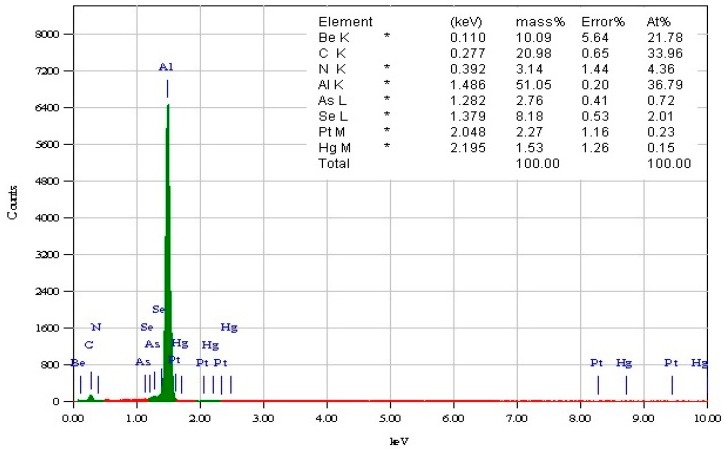
Energy dispersive X-ray spectroscopy (EDX) analysis of Al alloy showing the elemental composition.

The aluminum alloy workpiece material and the polycrystalline diamond (PCD) insert used as the cutting tool are shown in Figure 10. A commercially available aluminum alloy rod with an initial diameter of 6 mm was selected for the micro turning experiments. Energy dispersive X-ray spectroscopy (EDX) analysis was performed on the workpiece revealing its elemental composition. Figure 11 shows the EDX results (~51% Al by mass) of the workpiece material.

### 3.3. Cutting Force Measurement

**Figure 12:**
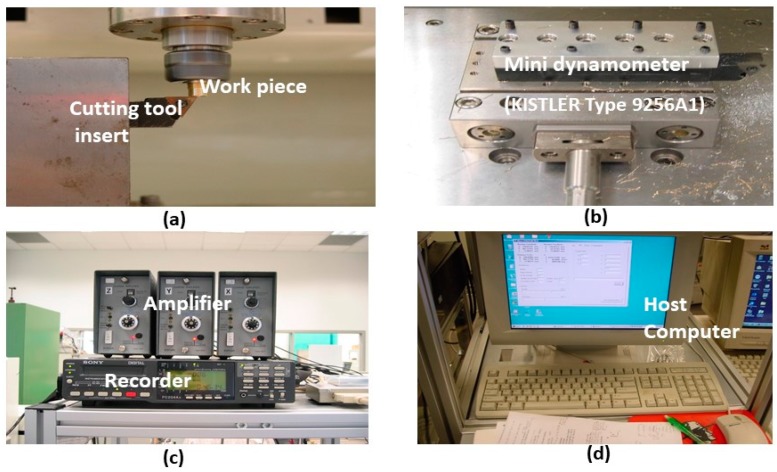
Experimental setup showing (**a**) cutting tool insert and the vertically oriented workpiece, (**b**) dynamometer with tool holder, (**c**) data recorder, and (**d**) host computer.

The Al alloy rod workpiece was fixed on the machine spindle. Machining was performed using a single point cutting tool (PCD insert). Mini dynamometer (KISTLER Type 9256A1, Kistler Group, Winterthur, Switzerland) attached to the cutting tool, measured the force signal during the micro cutting process. The force information, recorded with 24 kHz sampling frequency, was analyzed offline, after the experiment. The arrangement for force measurement is shown in Figure 12.

During the experiment, the variable parameters were spindle rotational speed (*s*), step length (*f*), and step size (*t*). The effects of the cutting parameters are explained in detail in Section 4.

## 4. Machining Results

The objective of this section is to obtain a suitable range of cutting parameters and their influence on the machining forces during the micro turning process.

### 4.1. Cutting Force Data

The measurement of cutting force components and the subsequent analysis provide important insight into the machinability factors of the aluminum alloy. Experiments were conducted by varying the step size (*t*), step length (*f*), and rotation (*s*). One parameter was varied while the other two were kept constant in order to identify the best combination of cutting parameters. The turning length was kept constant throughout, at 200 µm. The cutting conditions and corresponding measured force values are listed in Table 2.

#### 4.1.1. Influence of Step Size (*t*)

**Figure 13:**
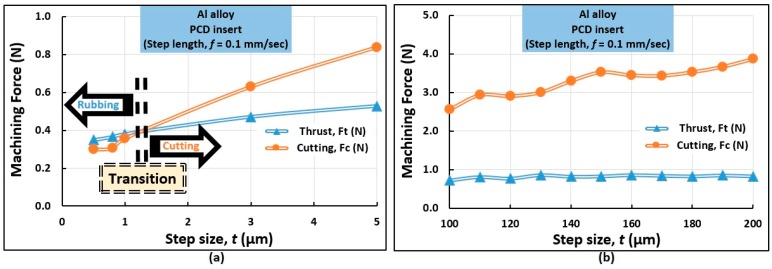
Influence of (**a**) smaller step size and (**b**) larger step size on the thrust and cutting forces in micromachining of Al alloy.

The influence of step size (*t*) on the thrust (*F*_t_) and cutting (*F*_c_) forces during the micro cutting of Al alloy at a rotation (*s*) of 1000 rev/min is plotted on Figure 13. At a shallow step size (*t* = 0.5 µm), the values of *F*_t_ and *F*_c_ were 0.35 N and 0.30 N, respectively. Thus, it can be inferred that the thrust force dominates over the cutting force when the step size is very small. This result is consistent with other examples of micro cutting in the literature [20]. In this regime, the mechanics of rubbing and burnishing is more influential than that of cutting. This is illustrated in Figure 13a. In contrast, increasing the step size (*t*) further causes the forces to elevate. *F*_c_ and *F*_t_ reached equality at around *t* = 1 µm. Thus, a “narrow transition regime” is observed for the progression from thrust to cutting forces, which is illustrated in Figure 13a. Subsequently, cutting forces exceed thrust forces as step size (*t*) is further increased. This is shown in Figure 13b. For example, at 200 µm step size, the values of *F*_t_ and *F*_c_ were found to be 0.83 N and 3.87 N, respectively. Thus, it can be inferred that at a larger step size (*t*), the cutting force dominates over thrust force akin to the cases of macromachining and conventional machining.

#### 4.1.2. Influence of Step Length (*f*)

**Figure 14:**
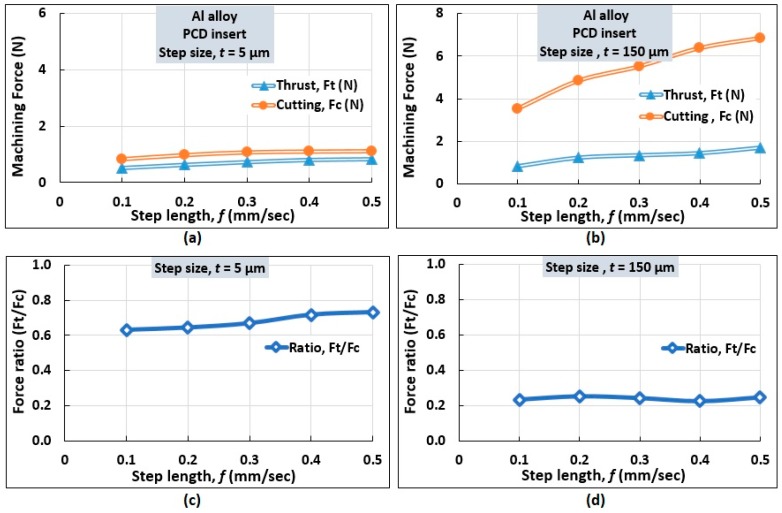
Influence of step length (*f*) on the (**a**,**b**) force and (**c**,**d**) force ratio (*F*_t_/*F*_c_) in the micro turning of Al alloy.

The influence of step length (*f*) on measured force components is identified graphically for two different step size (*t*) conditions while keeping the rotation (*s*) fixed at 1000 rev/min. This is shown in Figure 14a,b. At step size *t* = 5 µm, *F*_c_ was found to be the dominating force component. At *f* = 0.1 mm/s, the thrust and cutting force were 0.53 N and 0.84 N, respectively. An increase in step length (*f*) elevates the forces moderately. At *f* = 0.5 mm/s, the corresponding values of thrust and cutting forces were 0.82 N and 1.12 N, respectively. A similar trend was noted at a larger step size (*t* = 150 μm). However, a higher value of step length (*f*) rendered a higher cutting force than thrust force, as noticed in Figure 14b.

Moreover, the force ratio (*F*_t_/*F*_c_) was determined as shown in Figure 14c,d. For a small step size (*t* = 5 μm), it is noticed that *F*_t_/*F*_c_ increases with step length (*f*) as illustrated in Figure 14c. At a larger step size (*t* = 150 μm), as the step length is increased, a reducing trend was noticed similar to that observed in conventional machining [21].

#### 4.1.3. Influence of Rotation

**Figure 15:**
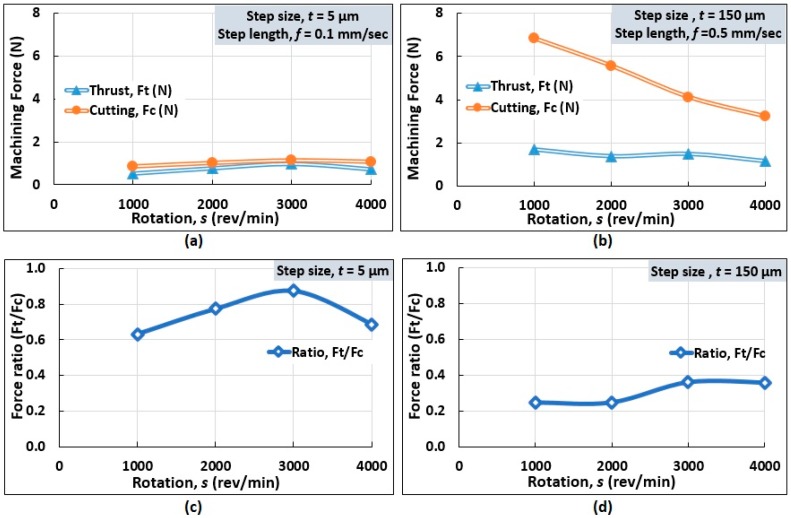
Influence of rotation on the cutting and thrust forces at (**a**) small step size and length, (**b**) large step size and length, and (**c**,**d**) the corresponding force ratios (*F*_t_/*F*_c_) in micro turning of Al alloy.

The influence of rotation on measured force components is depicted graphically in Figure 15. At a low step size and step length, the cutting force was found to be greater than thrust force, as shown in Figure 15a. At a rotation of 1000 rev/min, step size (*t* = 5 µm) and step length (*f* = 0.1 mm/s), the values *F*_c_ and *F*_t_ were 0.84 N and 0.53 N, respectively. Increasing the spindle rotation up to 3000 rev/min resulted in both the force components increasing linearly. This is due to the increased friction between the tool and the work material. With a further increment of the rotational speed, a slightly decreasing trend is observed because of reduced tool-workpiece contact area [22]. As for the force ratio (*F*_t_/*F*_c_), the value increased with an increasing rotational speed, before it maximized at 3000 rev/min. A subsequent increase in rotation resulted in a deflating force ratio, as shown in Figure 15c.

At a high step size and step length, the force components decreased with increasing rotations, as shown in Figure 15b. For example, at 1000 rev/min, the values of *F*_c_ and *F*_t_ were 6.84 N and 1.69 N, respectively, whereas at 4000 rev/min, cutting and thrust forces were 3.22 N and 1.16 N, respectively. An increase in speed caused a reduction in the material removal rate which reduced the cutting force owing to the reduced tool-Al alloy contact length [23]. Conversely, increasing rotations resulted in increasing force ratios (*F*_t_/*F*_c_) before plateauing at a rotational speed of 3000 rev/min, as depicted in Figure 15d.

These results, thus, demonstrate the thrust-dominated micro cutting mechanism at a small step size and the cutting-dominated mechanism for the macro cutting process at a large step size.

### 4.2. Chip Morphology

**Figure 16:**
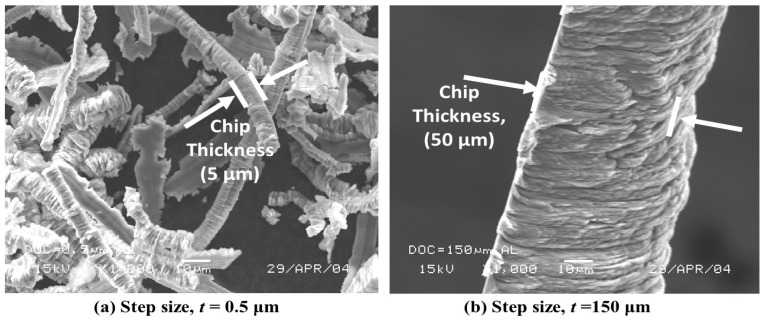
Variation of Al alloy chip thickness with step size (*t*).

During the chip formation process, the workpiece material is restricted from moving by the edge of the tool. The chip spreads sideways resulting in chip widths and thicknesses larger than the step size (*t*). The top surface of the chip exhibits corrugations. At a low step size (*t* = 0.5 μm), rubbing dominates over cutting between the cutting edge and workpiece surface. As a result, partly continuous and slice-type chips were observed with low step size. However, at an increased step size (*t* = 150 μm), thick chips were prevalent, as shown in Figure 16.

**Figure 17:**
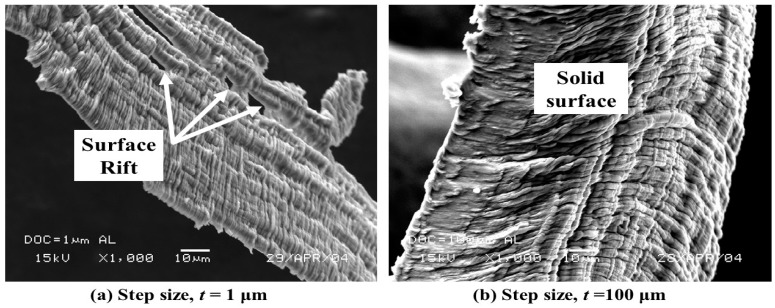
Variation of Al alloy chip surface morphology with step size (*t*).

At step size of *t* = 1 μm, rifted surfaces were noticed, as shown in Figure 17a. This is similar to that observed in shallow undeformed chip thickness in micromachining of Al alloy when the chip formation is governed by rubbing and ploughing [24]. At a large step size, *t* = 100 μm, the chip formation is dictated by shear deformation to produce the long and continuous chips [25], shown in Figure 17b.

**Figure 18:**
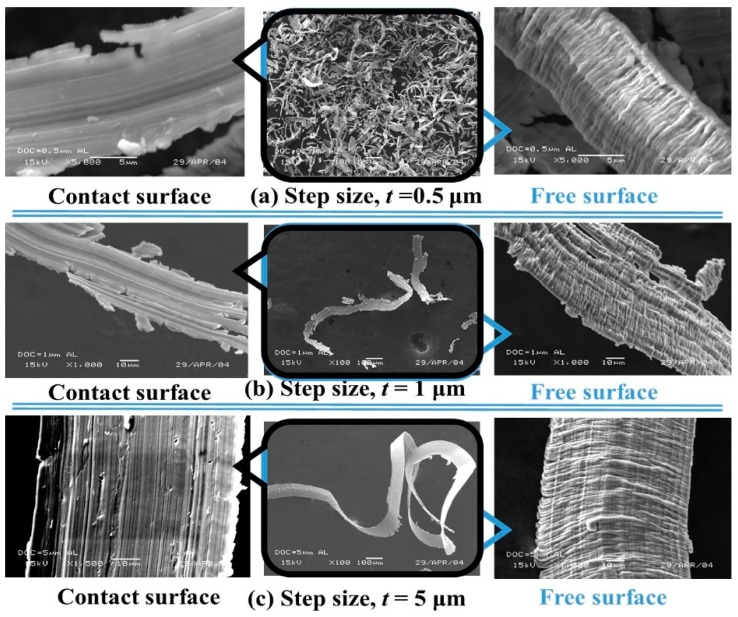
Influence of shallow step size (*t* = 0.5 to 5 μm) on the Al chip formation at step length *f* = 0.1 mm/s.

Figure 18 reports the types of chips that have been formed with the variation of step size (*t*). At a small step size (*t* = 0.5 μm), rubbing and ploughing were the governing mechanisms to form the chips of irregular shapes. The top surface or unrestricted surface of the chip, at step size *t* = 1 μm, showed quasi lamella or folding structure at 1000× magnification in scanning electron microscopy (SEM). In contrast, the bottom or restricted chip surface showed a smoother appearance, as shown in Figure 18b. Interestingly, both the top and bottom surfaces of the chip at step size *t* =1 μm showed similarities with rifts on the surface. With a higher step size (*t* = 5 μm), the folds on the top surface were found to be closely packed, however, the restricted surface showed a relatively smooth surface, as shown in Figure 18c, due to the tool-work contact.

**Figure 19:**
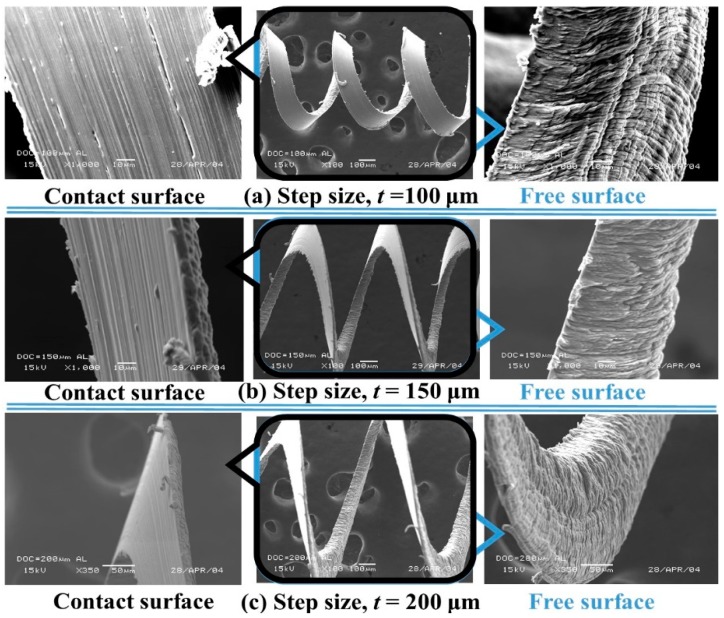
Influence of deep step size (*t* = 100–200 μm) on the Al chip formation at step length, *f* = 0.1 mm/s.

Figure 19a–c shows the SEM images of chips produced at even higher step sizes (*t*). Under these three conditions, the contact surface became smoother while the free surface exhibited corrugation. Here, an increased step size (*t*) resulted in the formation of curly and long continuous chips, similar to that produced in conventional machining and macromachining.

**Figure 20:**
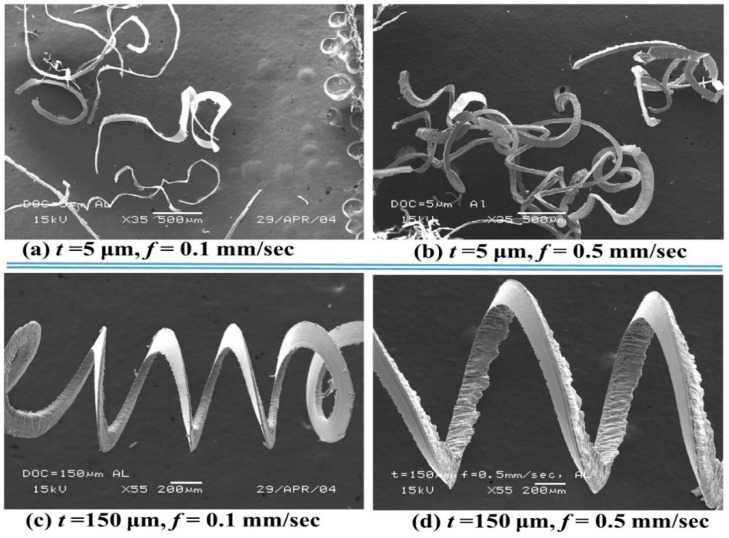
Scanning electron microscopy (SEM) micrograph of chips at different step lengths.

SEM observations of the chips formed from the micro turning of the aluminum alloy with a PCD tool indicated the ductile chip formation under different step lengths or feeds, as shown in Figure 20. With an elevated step length (*f*) or feed, the increased tool-work contact resulted in the formation of regular curly chips. As the step length (*f*) increased from 0.1 mm/s to a high value of 0.5 mm/s, at step size of 5 µm and speed of 1000 rev/min, the chip curls became more prominent, as observed in Figure 20a,b. However, at a higher step size (*t* = 150 µm), the increment of step length (*f*) resulted in long helical chips, as observed in Figure 20c,d.

## 5. Manufacturing Process of Microscopic Rods

Al alloy microrods of straight, tapered, and grooved end structures were fabricated using the micro turning process by applying the step cutting mechanism described in Section 2. The results of the fabrication process are described in detail in this section.

### 5.1. Determination of Step Length (f)

**Figure 21:**
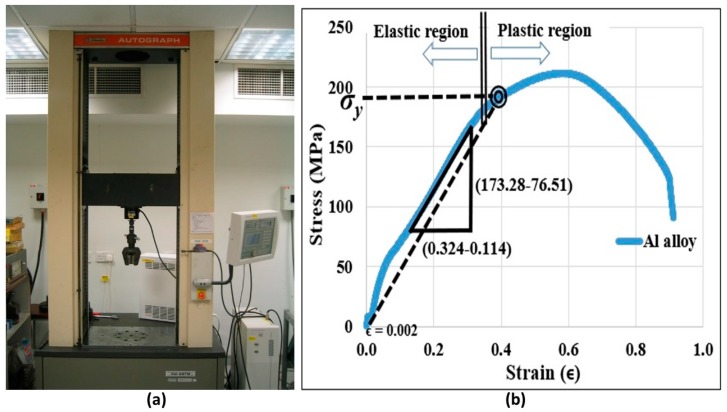
(**a**) Shimadzu AG-25TB tensile test machine and (**b**) stress vs. strain graph of Al alloy.

The step length (*f*), at which the permanent deformation of the aluminum alloy microrod is avoided, can be calculated from Equations (1) and (2). The Young’s modulus (*E*) and yield strength (*σ_y_*) necessary for the calculation were obtained from a tensile test conducted on a Shimadzu AG-25TB machine (Shimadzu Corporation, Kyoto, Japan). The 6 mm diameter aluminum alloy rod was pulled with tensile force increments of 0.625 kgf per 50 ms, until fracture. Figure 21a shows the machine and Figure 21b shows the stress-strain graph from the test. The Young’s modulus (*E*) was determined from the slope of the linear part of the stress-strain curve. The yield strength (*σ_y_*) was determined through the offset method by drawing a line parallel to the slope that begins at ϵ = 0.02% or 0.002. This is shown in Figure 21b.

Young’s modulus (*E*) = (173.28 − 76.51)/(0.32 − 0.114) = 461 MPa

0.02% offset yield strength (*σ_y_*) = 180 MPa

The deflection and stress on the workpiece were calculated as follows:

Using the experimental value of the thrust force *F*_t_ = 0.2246 N, when machining a rod of 0.2 mm diameter (*d*), the maximum step length (*f*) that can be actioned without causing plastic deformation can be calculated using Equation (2).

(4) $$\begin{array}{c} 180\times 10^{6}<\frac{32\times 0.2246f}{\pi \times {\left(0.2\times 10^{-3}\right)}^{3}}\\ f\;<\;0.63\;\mathrm{mm} \end{array}$$

To minimize the deflection of the rod tip, a small value of step length, *f* = 0.1 mm, is chosen. The corresponding deflection can be calculated for this step length using Equation (1).

(5) $$\delta =\frac{64\times 0.2246\times {\left(0.1\times 10^{-3}\right)}^{3}}{3\times \pi \times \left(461\times 10^{6}\right)\times {\left(0.2\times 10^{-3}\right)}^{4}}=2.068\;\mu m$$

Thus, for the step length and rod diameter chosen above, the corresponding stress acting on the rod is calculated using Equation (2).

(6) $$\sigma =\frac{32\times 0.2246\times {\left(0.1\times 10^{-3}\right)}^{3}}{\pi \times {\left(0.2\times 10^{-3}\right)}^{3}}=28.6\;\mathrm{MPa}\;\ll \;\sigma_{y}\;\left(180\;\mathrm{MPa}\right)$$

Therefore, the 0.2 mm diameter (*d*) rod will not permanently deform for the step length, *f* = 0.1 mm.

### 5.2. Microrod Fabrication

**Figure 22:**
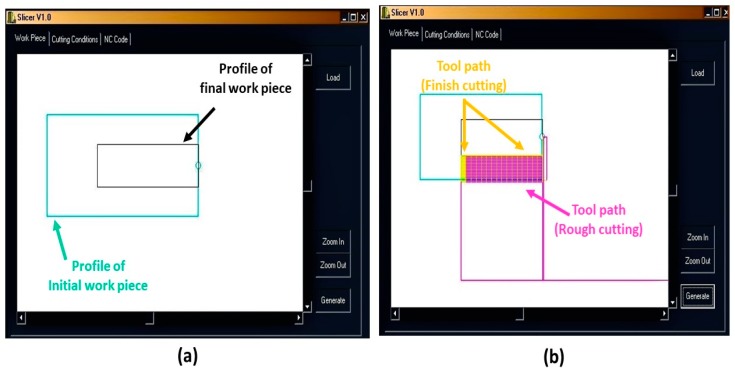
(**a**) Loading workpiece profile and (**b**) tool path of the cutting scheme.

The workpiece (aluminum alloy of 3 mm length and 1.5 mm diameter) and cutting tool (PCD) were attached to the main spindle and tool shank, respectively. The graphical user interface of the step cutting program (SLICER) is shown in Figure 6. The workpiece profile, before and after the micro turning process, is shown in Figure 22a. The simulated tool path from the input of the appropriate cutting conditions for the step cutting process is shown in Figure 22b. Upon the verification of the tool path, a CNC program was generated for the step cutting scheme from the selected cutting parameters. The NC program was subsequently uploaded to the user interface of the micromachining center (Figure 9) to run the program and perform the machining operation.

**Figure 23:**
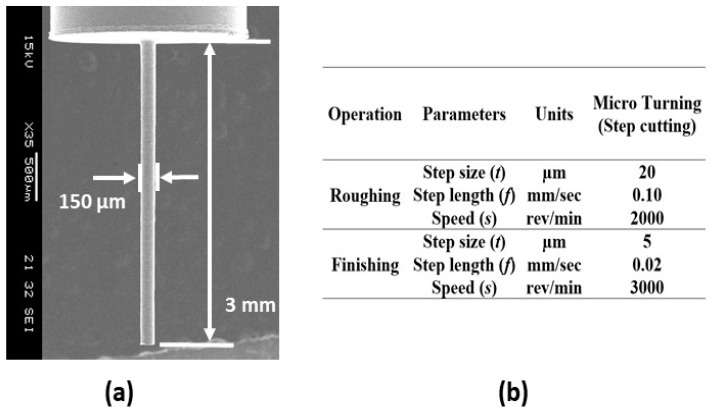
(**a**) Straight μ-rod and (**b**) machining parameters.

The cutting parameters were selected based on the micro turning of the aluminum alloy, as described in Section 4. Adequate measures were taken to keep the reaction forces as low as possible. Figure 23a shows the SEM image of the fabricated 150 µm diameter and 3.0 mm long shaft. Cutting parameters for this microfabrication process are provided in Figure 23b. It can be seen that the step length for the finishing operation was kept at a small value of 0.02 mm/s to minimize the reaction forces.

**Figure 24:**
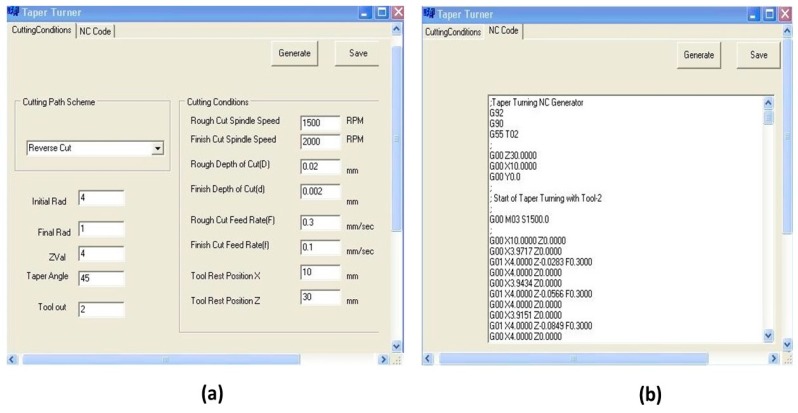
Graphical user interface (GUI) (**a**) developed for taper turning (**b**) of NC codes sample generated by the reverse cutting scheme.

The graphical user interface of the taper turning program, which is used to generate the NC codes for the micro taper turning operation, is shown in Figure 24. From the dimensions of the workpiece and appropriate machining parameters, the taper turning NC codes are generated according to the cutting path schemes (forward or reverse cut). Finally, the generated NC codes (CNC program) are uploaded to the machine controller to run the machining operation. Figure 24b shows a screenshot of sample NC codes for the groove cutting operation on the microrod.

**Figure 25:**
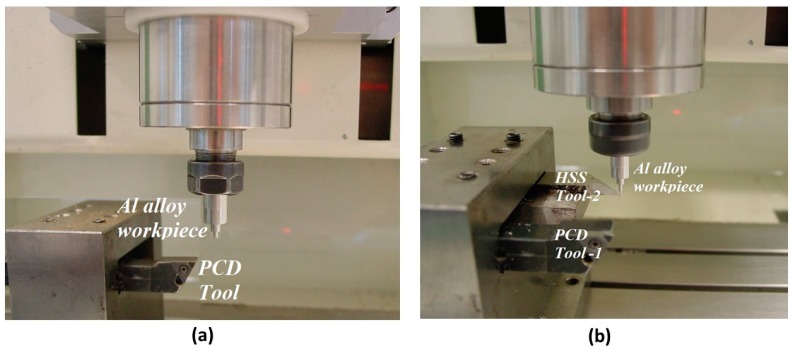
Micro taper turning setup for (**a**) forward cutting and (**b**) reverse cutting scheme.

The setup for the micro taper turning operation (both for forward and reverse cutting) is depicted in Figure 25. For the manufacturing of the conical tip rod, the taper micro turning scheme, as shown in Figure 25a, was adopted. Here, used a commercially available PCD insert, which can be seen affixed to the tool shank.

**Figure 26:**
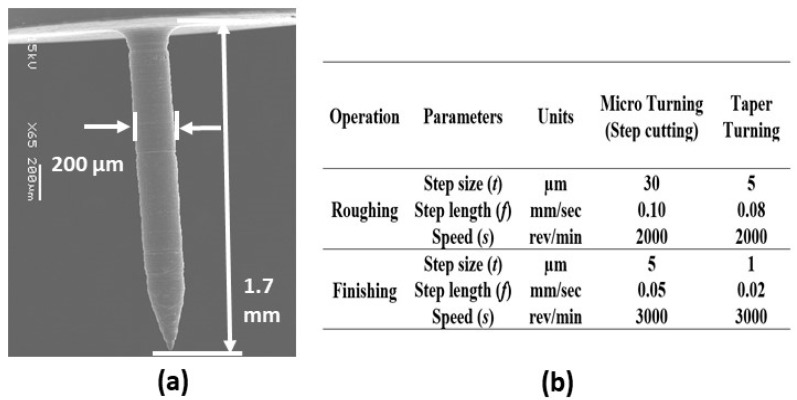
A fabricated 15° conical tip rod (**a**) diameter 200 μm and length 1.7 mm and (**b**) machining parameters.

A conical tip rod of diameter 200 μm was successfully fabricated using the machining parameter shown in Figure 26b. The length of the pin measured 1.7 mm and the taper angle was α = 15°.

**Figure 27:**
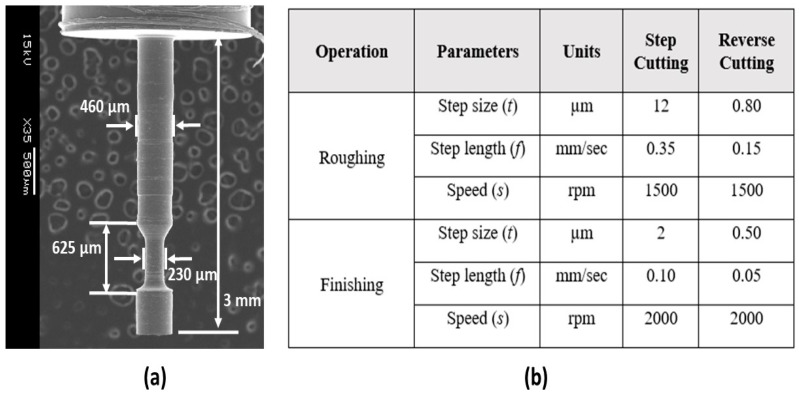
(**a**) Grooved microrod and (**b**) machining parameters.

For the manufacturing of the grooved rod, the taper micro turning with the reverse cutting scheme was used. The grooved microrod is shown in Figure 27a. Here, the right-hand tool (Tool-1), utilized for forward cutting, was a PCD insert attached to the tool shank. The left-hand tool (Tool-2), utilized for reverse cutting, was a high-speed steel (HSS) form tool. Before starting the machining process, two different coordinates were uploaded to the user interface for these tools. Figure 27a shows the grooves created on the microrod using the reverse cutting process. The optimal cutting conditions used for reverse cutting are listed in Figure 27b.

## 6. Conclusions

In this study, micro turning, a basic tool-based micromachining technology, was adopted to manufacture miniature components. Instructions to the machine, in the form of an NC program, were provided to control the micron range movements of the machine axes. Both SLICER and TAPER TURNER programs were used to generate the NC codes for straight and taper micro turning operations to fabricate Al alloy microrods of three different shapes. The following conclusions, thus, were drawn from this study:

- The deflection (*δ*) of the microrod, a major issue in micro turning, has been addressed by the step cutting scheme. The appropriate step length (*f*) was computed from the strength of Al alloy material to avoid permanent rod deformation;
- The step size (*t*) is an important parameter, which influences the cutting force components (*F*_c_ and *F*_t_) on the tip of the microrod. At a small step size (*t*), *F*_t_ was found to be greater than *F*_c_ due to the rubbing and burnishing action of the tool. However, the reverse phenomenon was observed at a larger step size (*t*) where *F*_t_ was the main force component;
- The SEM observation revealed the mechanisms of microchip formation for different cutting parameters. At a small step size (*t*), partially continuous microchips were observed. An interesting phenomenon in the form of a rifted chip surface was noticed in micromachining of Al alloy due to the governance of the rubbing and ploughing mechanism. At higher step sizes (*t*), curly and long continuous chips, similar to that produced in conventional machining and macromachining were noticed;
- Finally, micro turning was implemented to manufacture microfeatures on milli-scale structures. Microrods with straight, conical, and grooved tips were fabricated using an Al alloy material;
- A sharp single crystal diamond (SCD) tool can be used for the fabrication of the grooved microrod through the reverse cutting process as the HSS form tool (Tool-2) wears away quickly.

## Figures and Tables

**Table 1:** Manufacturing processes for producing μ-rods.

Process	Advantage	Disadvantage
Electrochemical Etching [11]	Minute shapes	Difficulty in dimension control
Micro turning [3]	Defined dimensions	High force can deform μ-rod
Micro grinding [12]	Electrical conductivity does not affect the process	The grinding force causes deformity
EDM [12]	No contact force	Slow process with shape limitation
Electroforming [13,14]	Complex parts with close tolerances	Limited material options

**Table 2:** Al alloy machining results.

Exp.	Machining Parameters			Measured Force Component	
Run No.	Step Size, *t* (µm)	Step length, *f* (mm/s)	Rotation, *s* (rev/min)	Thrust, *F*_t_ (N)	Cutting, *F*_c_ (N)
1	0.5	0.1	1000	0.35	0.30
2	0.8	0.1	1000	0.37	0.31
3	1	0.1	1000	0.38	0.36
4	3	0.1	1000	0.47	0.63
5	5	0.1	1000	0.53	0.84
6	5	0.1	2000	0.78	1.01
7	5	0.1	3000	0.99	1.13
8	5	0.1	4000	0.73	1.07
9	5	0.2	1000	0.63	0.98
10	5	0.3	1000	0.72	1.08
11	5	0.4	1000	0.80	1.11
12	5	0.5	1000	0.82	1.12
13	5	0.5	2000	1.63	2.17
14	5	0.5	3000	1.69	2.07
15	5	0.5	4000	1.57	1.84
16	10	0.1	1000	0.65	1.38
17	20	0.1	1000	0.68	1.47
18	30	0.1	1000	0.70	1.59
19	40	0.1	1000	0.70	1.77
20	50	0.1	1000	0.70	2.05
21	60	0.1	1000	0.83	2.27
22	70	0.1	1000	0.72	2.29
23	80	0.1	1000	0.77	2.29
24	90	0.1	1000	0.74	2.57
25	100	0.1	1000	0.72	2.56
26	110	0.1	1000	0.81	2.94
27	120	0.1	1000	0.77	2.91
28	130	0.1	1000	0.86	3.01
29	140	0.1	1000	0.82	3.30
30	150	0.1	1000	0.83	3.53
31	150	0.1	2000	0.98	2.70
32	150	0.1	3000	0.88	1.99
33	150	0.1	4000	0.81	1.81
34	150	0.2	1000	1.23	4.84
35	150	0.3	1000	1.34	5.51
36	150	0.4	1000	1.44	6.37
37	150	0.5	1000	1.69	6.84
38	150	0.5	2000	1.38	5.57
39	150	0.5	3000	1.49	4.12
40	150	0.5	4000	1.16	3.22
41	160	0.1	1000	0.86	3.45
42	170	0.1	1000	0.85	3.44
43	180	0.1	1000	0.83	3.53
44	190	0.1	1000	0.85	3.67
45	200	0.1	1000	0.83	3.87
